# Delivery and adjuvant: liposomes for SARS-CoV-2 vaccines

**DOI:** 10.5114/bta/207680

**Published:** 2025-08-26

**Authors:** Indira Putri Negari, Azka Narari Khoerunnisa, Tarwadi, Anissa Nofita Sari, Tsung-Hsien Chuang

**Affiliations:** 1 Research Center for Vaccine and Drugs, National Research and Innovation Agency (BRIN), Bogor, West Java, Indonesia; 2 Department of Biology, Faculty of Military Mathematics and Natural Sciences, Republic of Indonesia Defense University, Bogor, West Java, Indonesia; 3 Immunology Research Center, National Health Research Institutes, Zhunan, Miaoli, Taiwan

**Keywords:** liposome, drug delivery, vaccine, adjuvant

## Abstract

The global COVID-19 pandemic has highlighted the critical role of vaccines in controlling infectious diseases, with liposome-based formulations emerging as a pivotal advancement in vaccine technology. Liposomes are spherical vesicles composed of lipid bilayers that serve as drug delivery systems and versatile adjuvants, enhancing vaccine efficacy through improved antigen stability, targeted delivery, and immunogenicity. This review explores the potential of liposomes as adjuvants in both mRNA and protein subunit SARS-CoV-2 vaccines, detailing their composition and dual impact on innate and adaptive immune responses. Notably, liposome-based mRNA vaccines, such as those developed by Pfizer and Moderna, have demonstrated high efficacy by utilizing lipid nanoparticles to encapsulate mRNA and stimulate antigen-presenting cells, thereby inducing robust immune responses. Despite their advantages, challenges remain, including the optimization of lipid compositions and the mitigation of adverse immune effects. This review also examines the broad applications of liposomes in nanomedicine — from cancer therapy to antifungal treatments — and their potential for future vaccine development. By bridging the gap between engineering and immunology, the study of liposomes underscores their transformative potential in addressing current and emerging global health challenges.

## Introduction

Since the emergence of the SARS-CoV-2 virus, which caused the global COVID-19 pandemic in late 2019, numerous vaccines have been developed to combat the spread of this infectious agent worldwide. According to Viana et al. (2022), approximately 10 billion doses of COVID-19 vaccines had been distributed globally as of March 2022. Vaccination influences the body’s innate and adaptive immune responses in specific ways, inducing protection through the formation of immunological memory (Vetter et al. 2018).

To date, COVID-19 vaccines can be classified into four categories based on their platforms: whole virus vaccines, protein-based vaccines, viral vector vaccines, and nucleic acid vaccines (Ndwandwe and Wiysonge 2021). Among whole virus vaccines, inactivated or live attenuated types are the most common in the industry. Inactivated vaccines can be produced using several inactivation methods, including gamma rays, ultraviolet light, and formaldehyde treatment (Khoshnood et al. 2022). Because these vaccines contain the entire attenuated pathogen, they typically induce a stronger immune response and more effective lymphocyte stimulation than other vaccine types.

However, nucleic acid vaccines, such as DNA vaccines, also show great promise due to several advantages, including ease of large-scale production, the ability to stimulate both cellular and humoral immunity, and low production costs (Silveira et al. 2021). Although promising, the immunogenicity of DNA vaccines is generally lower than that of other platforms when administered *in vivo*, mainly due to suboptimal cellular uptake and limited plasmid delivery to antigen-presenting cells (APCs) as a result of DNA degradation (Eusébio et al. 2021). To address this limitation, adjuvants are often incorporated to enhance the immunogenicity of DNAbased vaccines (Narayanan et al. 2022).

Despite their limitations, mRNA vaccines – part of the nucleic acid vaccine category – along with viral vector vaccines, have been approved for COVID-19 (Yang et al. 2023). According to Doan et al. (2022), Virofree, a herbal medicine, has also shown potential in treating SARS-CoV-2 by targeting various viral entry and replication stages in the Delta and Omicron variants. Although these vaccines exhibit high efficacy due to their antigen components, nearly all depend on the inclusion of an adjuvant (Facciolà et al. 2022).

An adjuvant is a substance added to a vaccine to enhance and stimulate the immune response more effectively (Pulendran et al. 2021). Adjuvants work by improving antigen uptake by APCs, thereby activating antigen-specific immune responses and enhancing vaccine immunogenicity and efficacy, particularly in neonates, immunocompromised individuals, and the elderly (Mohan et al. 2013).

Adjuvants play a critical role in vaccine development and are typically classified based on their mechanism of action as either immune potentiators or delivery systems. Immune potentiators stimulate innate immunity via pattern recognition receptors such as Toll-like receptors (TLRs) and NOD-like receptors, or through cytokine signaling, whereas delivery systems aid in transporting antigens into immune cells (Facciolà et al. 2022; Fan et al. 2022; Haensler 2010; Zhao et al. 2023). Although immune potentiators show promise, particularly in tumor immunotherapy, they are often associated with toxicity and systemic side effects due to rapid diffusion into circulation (Abhyankar et al. 2021).

As a result, delivery-based adjuvants – such as aluminum salts, emulsions, liposomes, and polymers – are preferred for their improved safety profiles (Alving et al. 2016; Facciolà et al. 2022; Huang et al. 2024; Zhao et al. 2023). Among licensed adjuvants, aluminum salts and emulsions have proven effective for pathogen-based vaccines but are insufficient for diseases like malaria, tuberculosis, and acquired immunodeficiency syndrome (AIDS), which require robust cellular immune responses. These adjuvants may also lead to systemic reactogenicity, including fever, inflammation, and pain (Fan et al. 2022; Huang et al. 2024; Mohan et al. 2013; Pulendran et al. 2021).

In contrast, liposomes have emerged as a safe and effective adjuvant platform. They offer excellent biocompatibility, structural adaptability, high antigen encapsulation efficiency, and protection from degradation. Liposomes are also suitable for various routes of administration and enhance immune responses through targeted delivery, lysosomal release, and antigen cross-presentation (Nsairat et al. 2022; Tretiakova and Vodovozova 2022). Besides liposomes, other lipid nanoparticle (LNP) systems such as cationic lipids consisting of 1,2-di-O-octadecenyl-3-trimethylammonium propane (DOTMA) often combined with phospholipid or polymers to form stable complexes (lipoplexes) and improve encapsulation efficiency, cellular uptake, as well as endosomal escape – critical barriers in nucleic acid therapeutics (Hou et al. 2021). However, these LNPs systems are often limited by higher toxicity, poor stability, lower nucleic acid loading, and manufacturing complexity (Tada et al. 2015). Thus, other lipid adjuvants are needed for a superior balance of safety, efficacy, and scalability as the preferred platform for clinical drug delivery. One of the most common lipid adjuvants can fulfill these criteria: liposomes (Milicic et al. 2012).

**Figure 1:**
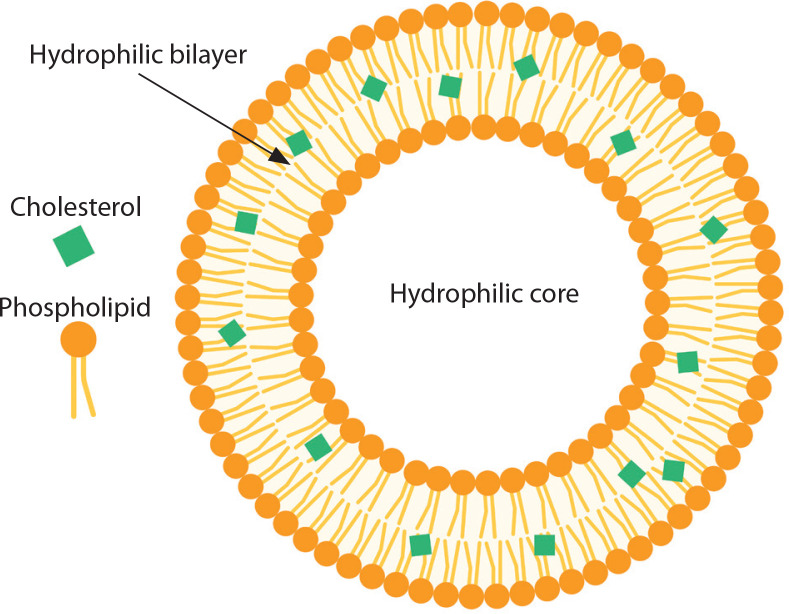
Schematic structures of liposomes. Created in https://BioRender.com

Liposomes are spherical, lipid-bilayer vesicular structures that enclose various compartments, as illustrated in Figure 1. Extensive studies have been conducted on liposomes as drug delivery systems due to their encapsulation capacity, which helps reduce antigen degradation and preserve a wide range of molecules. Additional advantages of liposomes include their ability to improve drug delivery stability and enhance efficacy by targeting specific cells and tissues (Henriksen-Lacey et al. 2011; Orosco and Espiritu 2024).

Furthermore, several liposomal characteristics – such as nanoparticle size, surface charge, bilayer rigidity, lipid composition, and preparation method – play critical roles in shaping the immune response to the target antigen (Nordly et al. 2009; Nsairat et al. 2022; Yanasarn et al. 2011). In addition to their established function in drug delivery, approximately 15 clinically licensed liposomal products are currently in use (Perrie et al. 2017).

Krasnopolsky and Pylypenko (2022) also reported the use of licensed liposomes and LNPs as adjuvants in vaccines, two of which are the Pfizer-BioNTech and Moderna COVID-19 vaccines. These formulations include LNPs composed of ionizable lipids, polyethylene glycol–lipid (PEG–lipid), 1,2-distearoyl-sn-glycero-3-phosphocholine (DSPC), and cholesterol. Specifically, the Moderna formulation includes ionizable lipid, DSPC, cholesterol, and 2-dimyristoyl-rac-glycero-3-methoxypolyethylene glycol- 2000 (PEG-2000-DMG).

Therefore, liposomes have been demonstrated to serve as a drug delivery tool and a vaccine adjuvant in the last decade of studies. Herein, the development and potential clinical use of liposomes in vaccines against COVID-19, as well as their specific effect on the immune system, are reviewed.

### Liposomes: from drug delivery to adjuvant

Liposomal formulations as drug delivery systems are currently undergoing clinical trials, with several already approved for use. In recent years, research has increasingly focused on the immunogenicity of liposome-based adjuvants in vaccines and on enhancing immune responses through liposome modifications. To understand these applications, it is essential first to examine the sources and composition of liposomes.

### Liposome compositions and applications

The term “liposome” is derived from two Greek words: “lipos” means fat, and “soma” means body. Dr. Alec D. Bangham, a British hematologist at the Institute of Cambridge, England, introduced the term in 1961 (Sharma and Agrawal 2021). In the 1970s, G. Gregoriadis proposed that liposomes could be used to deliver drugs to cell membranes, and their development has progressed rapidly since then (Gregoriadis 2016). Liposomes gained prominence due to their stability, encapsulation capability, and high drug-loading efficiency (Noble et al. 2014).

As shown earlier, liposomes are comprised of an area for an aqueous solution inside a hydrophobic membrane, making them capable of capturing drug molecules, including protein, peptides, carbohydrates, and DNA (Alving et al. 2016; Blanken et al. 2020; Boons 2010). Furthermore, their vesicle size, composition, and surface charge can be modified, providing flexibility for diverse medical applications (Zhou et al. 2023). These applications span from vaccines for viral, microbial, and fungal infections to cancer therapy (Huang and Anderson 2002; Orosco and Espiritu 2024).

Three key components contribute to the effectiveness of liposomes in biomedical and nanomedicine applications: (i) phospholipids, (ii) cholesterol, and (iii) PEG (Jiang et al. 2024).

Phospholipids typically consist of a hydrophilic headgroup, hydrophobic fatty acid chains, and a glycerol backbone (De Carvalho and Caramujo 2018). One of the most common sources of phospholipids is bacterial membranes (Kozhikhova et al. 2018). For instance, liposomes prepared from *Deinococcus radiodurans* and loaded into a respiratory syncytial virus vaccine were injected into mice and found to enhance vaccine efficacy (Huang and Anderson 2002). Halophilic bacteria have also been shown to offer strong potential for liposome production due to their ability to achieve high drug-loading efficiency (Baserisalehi 2020).

In addition to bacterial sources, several cationic lipids are well known for their gene delivery capabilities. These include 1,2-dioleoyl-3-trimethylammonium propane (DOTAP), 1,2-dioleoyl-sn-glycero-3-phosphoethanolamine (DOPE), and DOTMA (Ponti et al. 2021).

Chen et al. (2017) investigated a drug-in-cyclodextrinin-liposome (DCL) system for enhancing the delivery of lipophilic antitumor drugs. They encapsulated FITC-labeled hydroxypropyl-β-cyclodextrin (FITC-HP-β-CD) into liposomes composed of soybean-derived phosphatidylcholine (SPC) and modified with transferrin (Tf). The results showed that Tf-modified liposomes significantly improved stability and cellular uptake compared to PEGylated liposomes. SPC-based liposomes demonstrated the highest tumor cell internalization and lipophilicity, highlighting the potential of optimized Tf-DCL systems for targeted cancer therapy.

Other studies utilized microfluidics to optimize the production of monodispersed, drug-loaded liposomes for breast cancer treatment. Two types of PCs with varying acyl chain lengths were tested to control the release of doxorubicin hydrochloride. Liposomes were produced under optimized conditions (TFR 500 μl/min, FRR 0.1), resulting in six stable formulations (< 200 nm) with high encapsulation efficiency (> 80%) and sustained *in vitro* release. DMPC-based liposomes exhibited slower doxorubicin release than DSPC, and binary formulations showed higher cytotoxicity (IC_50_ ~1 μM) against MCF7, MDA-MB-231, and BT474 breast cancer cell lines, comparable to free doxorubicin. These findings support microfluidics as a robust platform for producing size-controlled liposomal nanomedicines for prolonged chemotherapeutic delivery (Gkionis et al. 2021). PC also showed potential in other drug delivery with thioether phosphatidylcholine (SPC-based) stealth liposomes as promising alternatives to conventional phospholipids for targeted, ROS-responsive drug delivery (Du et al. 2019). Serrano et al. (2015) demonstrated ascorbate PC liposomes for their preventive antioxidant and anti-inflammatory properties on whole human skin irradiated with UVA/UVB while allowing nonstable hydrophilic active ingredients to reach epidermis and dermis, preventing photodamage to the skin.

Another common phospholipid used in liposome formulations is phosphatidylethanolamine (PE), the second most abundant glycerophospholipid in eukaryotic cells. PE has recently gained attention due to its association with Alzheimer’s and Parkinson’s diseases (Calzada et al. 2016). Fan et al. (2017) investigated the pH-responsiveness mechanism of PE-based liposomes (PSLs), identifying them as key components in pHsensitive liposomal drug delivery systems for tumortargeted therapy.

In addition to PE, another study evaluated two radiotracers – ^18^F-duramycin (PE-targeting) and ^18^F-Zn-DPA (phosphatidylserine [PS]-targeting) – for their potential in apoptosis imaging, with possible clinical relevance in cancer diagnosis and therapy monitoring (Li et al. 2019). Regarding anticancer activity, other phospholipids such as sphingomyelin (SM) and cardiolipin (CL) have also shown promising therapeutic potential (Ahmadpour et al. 2020; Alrbyawi et al. 2022; Wang et al. 2024; Zhu et al. 2023). Sphingomyelin-based liposomes (SMLs) have notably advanced the development of lipid-based nanocarriers (Lim et al. 2021).

Meanwhile, CL, a signature phospholipid of mitochondrial membranes, is often linked to therapies for cardiac disorders (Gasanoff et al. 2021; Shen et al. 2015). Consequently, phospholipids play a vital role as the main structure of liposomes while also regulating the functional components inside liposomes (Jiang et al. 2024).

Cholesterol in cell membranes helps modulate the fluidity, stability, and permeability, sustaining the lipid bilayer’s rigid parts of the cell (Briuglia et al. 2015). The phospholipid structure is usually formed by four fused rings of hydrophobic lipids (Cerqueira et al. 2016). When the phospholipid’s hydrophobic tails interact with the nonpolar part of cholesterol and the polar part of cholesterol binds to the phospholipid’s hydrophilic headgroups, this results in a controlled release of molecules inside liposomes (Kaddah et al. 2018).

PEG is inherently hydrophilic, which imparts a “stealth” property to the surface of liposomes, helping them evade detection and degradation by the body’s immune system (Andra et al. 2022). In addition to prolonging the *in vivo* circulation time of liposomes, PEG as a polymer can enhance liposome stability (Shen et al. 2018). For example, Doxil^®^ was the first PEGylated liposomal formulation to encapsulate doxorubicin for anticancer treatment and has been approved for use against Kaposi’s sarcoma, breast cancer, and multiple myeloma (Mohamed et al. 2019). Thus, PEG is considered a critical component in boosting the drug delivery potential of liposomes.

Liposome applications range from general drug delivery to targeted cancer therapy. For instance, to address glioblastoma multiforme (GBM), Shi et al. (2019) developed a dual-functionalized, thermosensitive liposomal system (DOX@P1NS/TNC-FeLP) co-loaded with doxorubicin (DOX) and superparamagnetic iron oxide nanoparticles (SPIONs). The surface was modified with a GBM-targeting peptide (P1NS) and an antibody (TN-C) to achieve targeted delivery, while an alternating magnetic field triggered localized drug release. This system successfully crossed an *in vitro* blood–brain barrier (BBB) model, demonstrated GBM-specific uptake and drug release, and inhibited proliferation of U-87 GBM cells without affecting healthy brain cells. These results support the potential of DOX@P1NS/TNC-FeLP as a promising platform for BBB-penetrating, targeted GBM therapy.

In another approach, Rodà et al. (2023) employed Raman spectroscopy (RS) to characterize functionalized liposomes designed for targeted drug delivery in neurological disorders such as glioblastoma and Alzheimer’s disease. Furthermore, a novel strategy by Chen et al. (2020) involved embedding stiff nanobowls inside the aqueous core of DOX-loaded liposomes (DOX@NbLipo). This modification improved liposome stability against plasma proteins and shear forces during circulation, reducing drug leakage, enhancing tumor targeting, and increasing antitumor efficacy.

Liposomes composed of SPC, cholesterol, and CL and loaded with levofloxacin have demonstrated antibacterial activity against *Mycobacterium tuberculosis* (Gaidukevich et al. 2016). Another study addressed the challenge of achieving high liposomal loading efficiency for antibiotics by evaluating three clinically relevant drugs – vancomycin hydrochloride, teicoplanin, and rifampin – with varying degrees of hydrophilicity (Gonzalez Gomez et al. 2019). Encapsulation techniques were assessed based on encapsulation efficiency, lipid requirements, and mass yield. Hydrophobic antibiotics such as teicoplanin and rifampin showed higher encapsulation efficiencies with specific methods, while the hydrophilic vancomycin exhibited no clear preference. The study also highlighted methodological biases introduced by different quantification approaches, recommending ultrafiltration and methanol bursting as more accurate alternatives. These findings provide valuable insights for optimizing liposomal antibiotic delivery and inform broader nanocarrier design strategies (Ferreira et al. 2021; Gonzalez Gomez and Hosseinidoust 2020).

Liposomes have also proven useful as antifungal drug carriers, particularly against infections caused by *Candida albicans, Cryptococcus neoformans*, and *Aspergillus fumigatus*. Ambati et al. (2019) improved treatment efficacy and reduced toxicity by utilizing amphotericin B-loaded liposomes coated with sDectin-2, a mannan-binding domain that targets fungal cell wall components. These targeted liposomes demonstrated significantly enhanced binding and antifungal activity compared to untargeted formulations, allowing for lower effective doses. This approach shows promise for developing broad-spectrum antifungal liposomal therapies.

Anidulafungin-loaded liposome nanoparticles have also shown antifungal activity against both planktonic and biofilm forms of *Candida albicans* (Vera-González et al. 2020). In a related study, Bezerra et al. (2020) investigated the antifungal potential of farnesol, a bioactive compound, delivered via liposomes against various *Candida* strains. Farnesol-loaded liposomes enhanced antifungal efficacy and more effectively inhibited fungal dimorphism than free farnesol. Moreover, liposomal farnesol synergized with fluconazole, whereas free farnesol in combination with fluconazole exhibited antagonistic effects. These findings highlight the potential of farnesol-loaded liposomes for antifungal drug development, though further research is needed to understand their influence on drug resistance. Besides its applications in the medical field, liposomes have also gained interest in the food and pharmaceutical industry through the development of garlic extract encapsulated within PC and oleic acid (OA) as an antifungal agent in wheat bread, showing its potential as a natural antifungal agent in bakery products (Pinilla et al. 2019).

In addition to liposome applications, Snyder et al. (2016) compared pain control in total knee arthroplasty (TKA) patients who did not receive a femoral nerve block. Participants were administered either an intraoperative injection of bupivacaine liposome suspension (EXPAREL) or a concentrated multidrug cocktail. The study found that patients in the multidrug cocktail group reported significantly higher pain levels on postoperative days 1 and 2 and experienced more adverse events than those in the bupivacaine liposome group. Moreover, the bupivacaine liposome group reported greater satisfaction with both pain control and overall experience.

Similarly, Sporer and Rogers (2016) investigated the use of liposomal bupivacaine for postoperative pain management following primary TKA. Their study demonstrated a reduced need for breakthrough pain medication, improved pain scores at 12 h, and earlier ambulation when compared to a combined femoral nerve block and periarticular bupivacaine injection. These results highlight liposomes as a promising therapeutic strategy in pain management (Ji et al. 2021).

Mennini et al. (2015) further explored this potential by developing both conventional and PEGylated liposomes to enhance the pain-relieving effects of opiorphin. Using a rat tail-flick test, they compared the antinociceptive effects of these formulations to free opiorphin and morphine solutions (all at 5 mg/kg). Conventional liposomes increased opiorphin’s area under the curve (AUC) by 28% compared to the free peptide. PEGylated liposomes yielded even greater improvements, with AUC values 80, 60, and 40% higher than those of free opiorphin, morphine, and conventional liposomes, respectively. Additionally, PEGylated liposomes extended the duration of analgesic effect by over 50%, likely due to improved drug protection and prolonged circulation time. These findings suggest that opiorphin-loaded PEGylated liposomes could serve as a promising alternative to morphine for pain management.

Liposomes have demonstrated tremendous potential in gene therapy due to their advantages, such as biocompatibility, biodegradability, and high encapsulation efficiency (Liu et al., 2020; Tseu and Kamaruzaman 2023; Zylberberg et al. 2017). As a promising strategy for treating neurodegenerative diseases, dual-functionalized liposomes – modified with Tf for BBB targeting and penetratin (Pen) for enhanced cellular penetration – were developed to improve gene delivery. BBB model studies confirmed the ability of these liposomes to cross the barrier and transfect neurons *in vitro*. In *in vivo* studies, PenTf-liposomes accumulated significantly in the brain (12%) without causing cellular or structural damage. These liposomes successfully delivered plasmid DNA encoding β-galactosidase and GFP, resulting in measurable gene expression in mouse brains. These findings underscore the promise of multifunctional liposomes as platforms for gene therapy targeting neurodegenerative diseases (dos Santos Rodrigues et al. 2018).

In a related development, liposome-based systems are emerging as effective delivery platforms for clustered regularly interspaced short palindromic repeats (CRISPR)/Cas9 gene-editing technology, offering improved flexibility and delivery efficiency (Zhen et al. 2017; Zhen and Li 2020).

Chen et al. (2017) introduced liposome-templated hydrogel nanoparticles (LHNPs) as a novel co-delivery system for Cas9 protein and guide RNA. When integrated with minicircle DNA technology, LHNPs outperformed commercial transfection agents such as Lipofectamine 2000 in gene delivery, including to brain tumors. Targeted CRISPR/Cas9 delivery against the *PLK1* gene significantly inhibited tumor growth and improved survival in mice, showing LHNPs’ potential as a flexible platform for cancer gene therapy.

In another study, a nano-liposomal carrier system encapsulating therapeutic Cas9 with single-guide RNA (sgRNA) ribonucleoprotein (Cas9-RNP) demonstrated strong potential to enhance gene-editing therapies for human liver diseases (Cho et al. 2019).

Lower cholesterol content in liposomes increases membrane fluidity and enhances drug transport across the stratum corneum (Soni et al. 2016). While plain liposomes are not ideal for transdermal drug delivery due to limited skin penetration, they are effective for cosmetic use as they tend to remain within the upper layers of the skin (Musielak et al. 2024; Soni et al. 2016). Larger liposomes (≥ 1000 nm) are typically confined to the stratum corneum, whereas smaller liposomes (≤ 600 nm) can penetrate deeper (Estanqueiro et al. 2016). Liposomal systems can also enhance drug absorption by adhering to the skin surface, destabilizing or fusing with skin lipids, and disrupting the stratum corneum to facilitate penetration (Ahmadi Ashtiani et al. 2016). These mechanisms enable liposomes to improve the delivery of active ingredients from cosmetic and skincare products into the skin.

Bochicchio et al. (2020) utilized a novel semi-continuous simil-microfluidic technique to produce nanoliposomes loaded with vitamin D_3_, K_2_, E, and curcumin – compounds known for their antioxidant and skin-healing properties but typically unstable and poorly absorbed. This method yielded stable, negatively charged vesicles (84–145 nm) with high loading and encapsulation efficiency, demonstrating promising potential for cosmetic and dermo-cosmetic applications.

In cosmeceuticals, liposomes serve dual roles: as carriers for active ingredients and as active agents themselves (Ahmadi Ashtiani et al. 2016). In conditions like eczema or dry, damaged skin, empty liposomes can interact with skin lipids, proteins, and carbohydrates, aiding skin repair and restoring the barrier function of the stratum corneum. As delivery systems, liposomes offer numerous advantages: enhanced penetration, solubility, and stability of active ingredients; extended therapeutic effects; protection against environmental degradation; targeted delivery; reduced toxicity; and improved pharmacokinetic and pharmacodynamic control – ultimately making products more cost-effective (Ahmadi Ashtiani et al. 2016; Hameed et al. 2019).

Liposomes have also been used to deliver vitamin D_3_ due to their stability and ability to enhance its effectiveness as a skin-protective agent against photoaging (Bi et al. 2019). However, many cosmetic actives used in novel formulations, such as sunscreens and anti-aging treatments, suffer from low penetration, poor solubility, and physicochemical instability (Kim et al. 2018). Lipid carriers like liposomes help address these issues by increasing solubility, improving skin permeation, and enhancing the stability of poorly soluble compounds (Pavlou et al. 2021).

Vovesná et al. (2021) developed stable liposomal formulations containing ceramides and other skin lipids to repair damaged skin barriers – often linked to reduced ceramide levels. Using lipid film hydration and high-pressure homogenization, liposomes were prepared with ceramides 3 and 6, cholesterol, stearic acid, and 10% urea in phosphate-buffered saline. When tested on chemically damaged porcine skin, this formulation significantly improved barrier function, reducing permeation to levels closer to those of intact skin. Notably, nonhomogenized liposomes were more effective than homogenized ones. FTIR analysis supported these results, highlighting the potential of liposomal therapies in skin barrier repair.

In another study, Kim et al. (2015) investigated the moisturizing effects of liposomal serine combined with different cosmeceutical bases. Serine, a key component of the skin’s natural moisturizing factors, was hypothesized to enhance hydration when effectively delivered to the stratum corneum. Among the four tested bases, hydrogel showed the best water-holding capacity. Incorporating 1% liposomal serine into the hydrogel significantly improved skin moisturization – 1.62 to 1.77 times more than hydrogel alone, free serine, or blank liposomes. The moisturizing effect was not dependent on serine concentration, supporting liposomal serine as a promising agent for advanced skincare formulations.

Finally, and importantly, liposomes have also been employed as platforms in theranostic applications to encapsulate both therapeutic and diagnostic agents (Xing et al. 2016). Theranostics refers to technologies that integrate therapeutic and diagnostic functions into a single system (Kim and Jeong 2021).

For example, Ren et al. (2019) investigated how the physical and chemical properties of liposomes influence their ability to passively target inflamed joints in rheumatoid arthritis (RA). Using a collagen-induced arthritis mouse model, the researchers evaluated liposomes with varying sizes, surface charges, and PEG modifications. The results indicated that liposomes with a 100 nm diameter, a slightly negative surface charge, and 10% 5 kDa PEG achieved optimal circulation time and selective targeting of RA-affected joints. When loaded with dexamethasone, these optimized liposomes significantly improved antiarthritic effects.

In addition to RA, liposomes have been widely studied for their roles in tumor and cancer-targeted therapies, including drug delivery to gliomas, chemotherapy enhancement, and cancer detection (Alavi and Hamidi 2019; Belfiore et al. 2018; Bozzuto and Molinari 2015; Mojarad-Jabali et al. 2021; Mukherjee et al. 2022; Pérez-Herrero and Fernández-Medarde 2015; Riaz et al. 2018; Wang et al. 2023).

### Liposome and LNPs applications in viral infections beyond SARS-CoV-2

Liposomes have also been employed in addressing viral infections and virus-associated diseases beyond SARS-CoV-2. Notably, their use has been explored in the treatment and prevention of HIV, where liposomal formulations enhance drug stability, improve targeted delivery to immune cells, and reduce systemic toxicity (Gao et al. 2018). In a related study, Leaman et al. (2021) developed broadly neutralizing antibodies (bNAbs) targeting the virus’s native, membrane-bound envelope glycoprotein (mEnv). Membrane Env liposomes (MELs) are a novel liposome-based platform that displays multivalent, structurally intact mEnv trimers. In this system, purified mEnv spikes are reconstituted onto naked liposomes using a detergent-removal method, resulting in stable nanoparticles (~133 nm) with proper antigen orientation. These MELs preserved native epitopes recognized by bNAbs while excluding non-neutralizing antibody (non-NAb) binding. When used in sequential immunization in rabbits, MELs successfully elicited antibodies capable of neutralizing tier 2 HIV isolates.

Additionally, plasma membrane-derived liposomes have demonstrated robust antiviral activity against herpes simplex virus type 1 (HSV-1), outperforming Chinese hamster ovary (CHO)-derived liposomes (Bhattacharya et al. 2022). Another study reported the development of decoy liposomes functionalized with heparan sulfate octasaccharide (HS-octa) as a broad-spectrum antiviral strategy against HSV, respiratory syncytial virus (RSV), and human parainfluenza virus 3 (hPIV3). The ability of liposomes to modulate and elicit adjuvant effects through diverse lipid combinations further supports their use in delivering anti-RSV agents (Joshi et al. 2019).

In another application, Croci et al. (2016) developed and evaluated liposome-based formulations of ivermectin – a potent antihelminthic drug shown to inhibit flavivirus helicases *in vitro*, though its clinical use is limited by poor solubility and high cytotoxicity. The engineered liposomes significantly reduced ivermectin’s cytotoxicity across multiple cell lines and enhanced its antiviral efficacy against several Dengue virus strains (1, 2, and S221). These results confirmed the antiviral potential of ivermectin and demonstrated the value of liposomes as effective drug carriers that improve pharmacokinetics and therapeutic outcomes. This liposomal approach offers a promising strategy for optimizing ivermectinbased antiviral therapies.

Hongtu et al. (2023) demonstrated a promising approach to rabies vaccination using an mRNA vaccine encoding the rabies virus glycoprotein (RABV-G), encapsulated in LNPs and combined with various nucleic acid-based immune stimulators, including CpG 1018, CpG 2395, and Poly I:C. Among these, the formulation with LNP and CpG 1018 elicited the strongest immune response, inducing high and sustained levels of RABV-G-specific IgG, potent virus-neutralizing antibodies, and robust cell-mediated immunity, including IFN-γ- and TNF-α-producing CD4^+^ and CD8^+^ T cells. Notably, this combination provided 100% protection in both pre- and post-exposure models and significantly reduced viral replication. These findings suggest that LNP-formulated RABV-G mRNA with CpG 1018 is a safe, effective, and scalable vaccine candidate that could serve as a next-generation alternative to traditional rabies vaccines. However, the success observed with SARS-CoV-2 vaccines has not been equally replicated in other antiviral activities of certain drugs or vaccines, partly due to the virus’s high antigenic variability, dense glycan shield, and the need for prolonged and sequential immunization to guide antibody maturation.

### Types of liposomes

Liposomes can be classified in several ways, including by size, lamellarity, and surface charge (González-Rodríguez and Rabasco 2011; Kraft et al. 2014; Liu et al. 2022). Based on size, liposomal nanoformulations typically range from 50 to 500 nm and are well-suited for nanomedicine applications (Lombardo et al. 2019). Smaller liposomes, particularly those under 50–100 nm, can evade immune detection, exhibit prolonged circulation in the bloodstream, and allow for enhanced drug release (Andra et al. 2022; Ren et al. 2019).

Another classification of liposomes is based on lamellarity, which is divided into: unilamellar vesicle (ULV), which contains only one bilayer membrane; oligolamellar vesicle (OLV), which has around 2–5 bilayer membranes; and multilamellar vesicles (MLV) with bilayer membranes resembling onion-like structures with many layers of membranes (Jiang et al. 2024; Pattni et al. 2015).

Lamellarity influences the drug release rate; the more bilayers present, the slower the encapsulated molecules are released (Lombardo et al. 2019). It is also important to note that both the size and lamellarity of liposomes can be controlled through preparation methods and modifications to their surface charge.

DOTAP is an example of a charged, cationic liposome that has been widely studied for its ability to deliver negatively charged macromolecules, such as DNA and RNA (Majzoub et al. 2016). Due to their positive surface charge, cationic liposomes attract negatively charged nucleic acids via electrostatic interactions, facilitating cellular uptake and enhancing drug delivery through easier diffusion across cell membranes (Ochoa-Sánchez et al. 2024). However, despite these advantages, cationic liposomes are often associated with high toxicity and low transfection efficiency (Neves et al. 2016).

In contrast, anionic liposomes are less toxic and typically exhibit shorter circulation times. Although they are less stable and have weaker binding affinity for nucleic acids, their biocompatibility makes them more suitable for transdermal rather than gene delivery applications (González-Rodríguez and Rabasco 2011). For instance, Ibaraki et al. (2019) explored the rigidity of anionic liposomes, such as 1,2-dioleoyl-sn-glycero-3-phospho-L-serine (DOPS), and demonstrated their potential in dermal drug delivery systems.

Beyond liposome classification, structure, and composition, LNPs are a critical determinant of performance in vaccine delivery systems (Nordly et al. 2009; Song et al. 2012). LNPs are typically composed of ionizable lipids, helper phospholipids, cholesterol, and PEG-lipids, each contributing distinct functional roles (Hou et al. 2021; Schoenmaker et al. 2021). Ionizable lipids aid in endosomal escape and antigen delivery by becoming positively charged in acidic environments, while cholesterol enhances membrane stability and maintains structural integrity (Cerqueira et al. 2016; Wang et al. 2024). Phospholipids such as DSPC or DOPE influence bilayer fluidity and promote fusion with immune cells (Ponti et al. 2021). PEG-lipids prolong circulation time and prevent aggregation; however, excessive PEGylation can hinder cellular uptake and reduce immunogenicity (Mohamed et al. 2019).

Fine-tuning the composition of these components enables LNPs to balance antigen protection, stability, and delivery efficiency — critical factors for optimizing vaccine performance.

### Licensed liposomal drug products

Since liposomes were first recognized as drug delivery systems in the 1970s, hundreds of nanodrug formulations have entered clinical development, with several already licensed for medical use (Gatto et al. 2024; Jiang et al. 2024). This widespread implementation demonstrates the versatility of liposomes as platforms for anticancer therapies, antifungal treatments, and vaccine adjuvants — applications that continue to expand, as summarized in Table 1.

**Table 1:** A summary of liposomal drug products approved by the U.S. Food and Drug Administration (FDA) as of 2024

Brand name	Year of approval	Liposomes formulation	API	Indication	Route of administration	References
Doxil^®^	1995	HSPC:CHOL:DSPE-PEG2000	Doxorubicin	Kaposi’s sarcoma, ovarian cancer, and myeloma	*i.v.*	Liu et al. 2022; Nsairat et al. 2022; Jiang et al. 2024; Gatto et al. 2024
DaunoXome^®^	1996	DSPC:CHOL	Daunorubicin	Kaposi’s sarcoma	*i.v.*	Liu et al. 2022; Nsairat et al. 2022; Jiang et al. 2024; Gatto et al. 2024
Amphotec^®^	1996	Amphotericin B:Cholesteryl sulphate	Amphotericin B	Fungal infection	*i.v.*	Jiang et al. 2024
AmBisome^®^	1997	Amphotericin B: HSPC:DSPG:CHOL	Amphotericin B	Fungal infection	*i.v.*	Liu et al. 2022; Nsairat et al. 2022; Jiang et al. 2024; Gatto et al. 2024
Inflexal^®^ V	1997	Lecithin:Cephalin:Phospholipids	Virus antigen	Influenza	*i.m.*	Jiang et al. 2024
DepoCyt^®^	1999	DepoFoam™	Cytarabine	Lymphomatus meningitis	Spinal	Liu et al. 2022; Nsairat et al. 2022; Gatto et al. 2024
Myocet^®^	2000	CHOL:EPG	Doxorubicin	Cancer	*i.v.*	Liu et al. 2022; Nsairat et al. 2022; Jiang et al. 2024
Visudyne^®^	2000	Verteporphin:DMPC with EPG	Verteporphin	Age-related degeneration	*i.v.*	Liu et al. 2022; Nsairat et al. 2022; Jiang et al. 2024; Gatto et al. 2024
DepoDur^®^	2004	DepoFoam™	Morphine sulfate	Painkiller	Epidural	Liu et al. 2022; Nsairat et al. 2022; Jiang et al. 2024; Gatto et al. 2024
Mepact^®^	2004	DOPS:POPC	Mifamurtide	Cancer	*i.v.*	Liu et al. 2022; Nsairat et al. 2022; Jiang et al. 2024; Gatto et al. 2024
Exparel^®^	2011	DepoFoam™	Bupivacaine	Anesthesia	*i.v.*	Liu et al. 2022; Nsairat et al. 2022; Jiang et al. 2024; Gatto et al. 2024
Marqibo^®^	2012	SM:CHOL	Vincristine	Leukemia	*i.v.*	Liu et al. 2022; Nsairat et al. 2022; Gatto et al. 2024
Onivyde^®^	2015	DSPC:CHOL:DSPE	Irinotecan	Pancreatic adenocarcinoma	*i.v.*	Liu et al. 2022; Nsairat et al. 2022; Jiang et al. 2024; Gatto et al. 2024
Vyxeos^®^	2017	DSPG:DSPC:CHOL	Daunorubicin and cytarabine	Leukemia	*i.v.*	Liu et al. 2022; Nsairat et al. 2022; Jiang et al. 2024; Gatto et al. 2024
Onpattro^®^	2018	CHOL, DLin-MC3-DMA: DSPC:PEG2000-C-DMG	Patisiran	Hereditary transtyretin-mediated amyloidosis	*i.v.*	Liu et al. 2022; Nsairat et al. 2022; Jiang et al. 2024; Gatto et al. 2024
Comirnaty^®^	2021	ALC-0315:ALC-0159: CHOL:DSPC	mRNA	COVID-19	*i.m*	Jiang et al. 2024; Gatto et al. 2024
Spikevax^®^	2022	SM-102:mPEG2000-DMG: CHOL:DSPC	mRNA	COVID-19	*i.m*	Jiang et al. 2024; Gatto et al. 2024

Among these licensed liposome products, anticancer drugs held the first place for having a great deal of quantity, concerning about seven marketed medicines, including the first liposome-approved product by the U.S. Food and Drug Administration (FDA) in 1995: Doxil^®^ for Kaposi’s sarcoma, ovarian cancer, and multiple myeloma treatment (Mohamed et al. 2019).

In the antifungal category, liposomal drugs such as Amphotec^®^ and AmBisome^®^ have been approved for clinical use (Jiang et al. 2024). These formulations encapsulate amphotericin B as the active pharmaceutical ingredient (API). Therefore, it suppresses the toxic activity from fungal infection (Nsairat et al. 2022).

The Inflexal^®^ V vaccine was the first licensed liposomal vaccine, formulated with two influenza virus strains (type A and type B) (Asadi and Gholami 2021; Krasnopolsky and Pylypenko 2022). Clinical trials of Inflexal^®^ V demonstrated a strong humoral immune response, with immunogenicity levels several times higher than existing influenza vaccines at the time (Poon and Patel 2020).

Liposomes’ high encapsulation capacity also allows them to carry macromolecules such as DNA and RNA, enhancing vaccine efficacy by functioning as adjuvants. During the 2019 COVID-19 pandemic outbreak, researchers leveraged the ability of liposomes to extend the half-life of antigens in the bloodstream, thereby ensuring prolonged exposure to APCs and promoting stronger immune responses (Krasnopolsky and Pylypenko 2022; Mohan et al. 2013).

**Figure 2:**
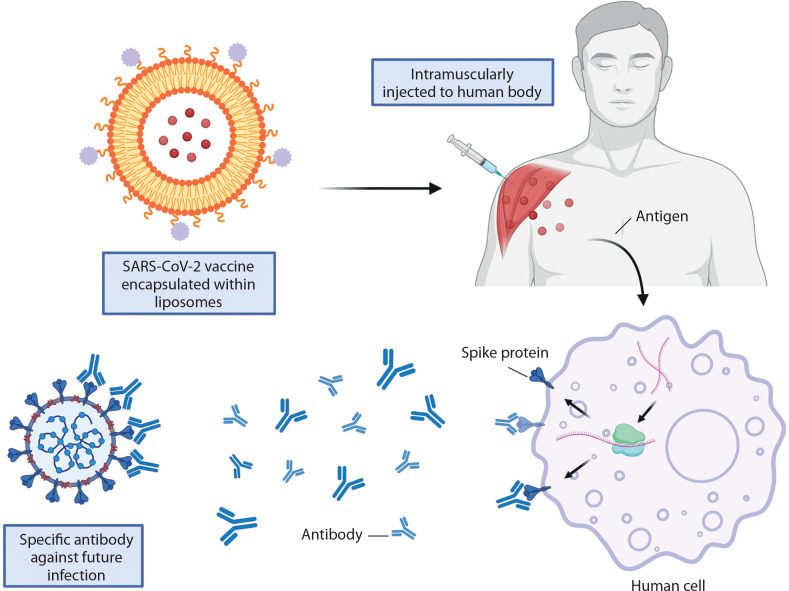
Liposome encapsulation ensures antigen protein translation in SARS-CoV-2 vaccines. Created in https://BioRender.com

This was exemplified by the Pfizer/BioNTech and Moderna vaccines, which utilized liposome-based formulations to develop mRNA vaccines against COVID-19. The primary components of these formulations include cationic lipids, PEGylated lipids, phospholipids, cholesterol, and mRNA encoding the viral spike glycoprotein in which the latter were encapsulated inside the liposomes to protect it from enzyme degradation for an efficient antigen protein translation after administered intramuscularly (Figure 2). Upon delivery into human cells, the mRNA is translated into viral spike proteins, triggering both innate and adaptive immune responses that result in localized inflammation (Schoenmaker et al. 2021). Leukocytes, including neutrophils and APCs, are recruited to the site of inflammation, leading to the generation of antibodies that prevent SARS-CoV-2 infection (Gregoriadis 2021).

By contemplating the biodegradability and versatility of liposomal formulations in vaccine fields, it is safe to say that further development of liposomes as another vaccine adjuvant will be looked forward to. Other than being a drug delivery system, liposomes have every potential and significant effect on the human immune system while also achieving synergism with the mRNAencoded proteins (Hou et al. 2021). Utilizing billions of vaccine doses against SARS-CoV-2 saved countless human lives due to the liposomal formulation and its immunogenicity (Cheng et al. 2021). Moreover, numerous liposomal vaccines are underway, ready to catch up as another licensed drug for medical applications.

### Immune system response to liposomes

Ongoing research has led to a growing number of licensed and approved liposome-based drug products each year. Two prominent examples that contributed to combating the COVID-19 pandemic are Spikevax^®^ (Moderna) and Comirnaty^®^ (Pfizer). Further studies are taking place regarding the liposomal formulation in vaccines and its impact on the body. While liposomes offer numerous advantages, they are not without drawbacks. Therefore, understanding their impact on the immune system is essential.

### Innate immunity and liposomes

When liposomes are introduced into the body, they are treated similarly to other foreign particles – that is, as antigens (Tretiakova and Vodovozova 2022). They are typically recognized and taken up by APCs through phagocytosis or receptor-mediated endocytosis. Innate immunity represents the body’s first line of defense and is mediated by macrophages, neutrophils, dendritic cells, eosinophils, and NK cells (Lee et al. 2023).

Liposome-based mRNA vaccines for COVID-19 significantly impact the innate immune response through the activation of pattern recognition receptors, such as TLRs, melanoma differentiation-associated gene 5 (*MDA5*), and NOD-, LRR-, and pyrin domain-containing protein 3 (NLRP3) (Newton and Dixit 2012; Takeuchi and Akira 2010). These receptors are capable of detecting mRNA encapsulated within LNPs, leading to the production of key cytokines – IL-1β, IFN-γ, and IL-6 – which are essential for initiating an antiviral state (Chen et al. 2018; Iwasaki and Medzhitov 2015).

**Figure 3:**
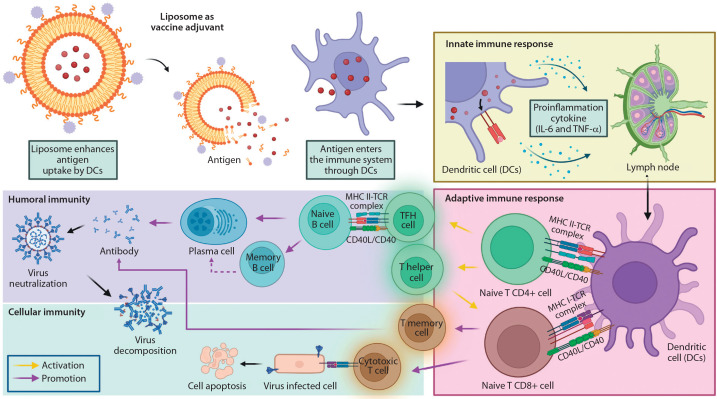
Immune response mechanisms following intramuscular vaccine injection of liposome as an adjuvant. Upon vaccination, antigens are presented on the surface of dendritic cells via major histocompatibility complex (MHC) molecules right after liposomes are ingested, producing inflammatory cytokines, thus called an innate immune response. Liposomes as adjuvants promote lymphocytes within the lymph node, which activate nad’ve CD4+ and CD8+ T cells. The process initiated an adaptive immune response. CD4+ and CD8+ T cells were the precursors of T follicular helper (TFH) cells, which promote B cell expansion and differentiation, while T helper cells assist in the activation of T memory cells as well as cytotoxic T cells, respectively. Created in https://BioRender.com

The delivery mechanism of liposomes further influences the innate immune response, as illustrated in Figure 3. Tahtinen et al. (2022) demonstrated that empty LNPs (eLNPs) alone can stimulate cytokine production, functioning as immune potentiators. This indicates that innate immune activation depends not only on the presence of mRNA but also on the lipid composition and vaccine formulation. For example, SM-102 LNPs were found to induce higher IL-1β secretion than other lipid formulations, emphasizing the role of specific lipid components in modulating immune responses.

However, excessive activation of these pathways may lead to systemic inflammation, cytokine storms, and hypersensitivity reactions, including anaphylaxis, which has been associated with PEGylated lipids (Risma et al. 2021). Additionally, the overproduction of type I interferons can suppress mRNA translation, thereby reducing vaccine efficacy (Crow et al. 2019).

### Adaptive immunity and liposomes

The adaptive immune response triggered by liposome-based mRNA COVID-19 vaccines involves strong activation of both T cells and B cells (Lee et al. 2023). These vaccines deliver mRNA encoding specific antigens, which are processed and presented by APCs, thereby promoting CD8^+^ T cell responses. For example, Keshari et al. (2024) investigated a neoantigen mRNA vaccine in preclinical models and demonstrated its ability to stimulate neoantigen-specific T cell responses.

Liposome-based mRNA vaccines also enhance the differentiation of T follicular helper (TFH) cells, which are critical for B cell activation and antibody production. Among the key cytokines involved in this process, IL-6 plays an essential role in TFH cell differentiation, ensuring a strong humoral immune response (Korn and Hiltensperger 2021; Li et al. 2018).

Furthermore, mRNA vaccines play a role in inducing memory responses that could create long-lasting immunity. The route of administration also plays a role in adaptive immunity since intramuscular injections promote systemic T and B cell activation. Meanwhile, intravenous administration demonstrates enhanced antigen-specific CD8^+^ T cell responses through RNA-lipoplex (RNA-LPX) vaccines, inducing robust activation of antigen-specific CD8^+^ T cells, promoting their expansion to comprise up to 60% of the total CD8^+^ population. These cells also exhibit full effector functions, including the production of IFNγ, TNFα, granzyme B, and expression of degranulation markers such as CD107a/b. Additionally, RNA-LPX vaccination supports forming memory CD8^+^ T cells capable of rapid recall responses upon antigen re-exposure.

Importantly, the acquisition of effector function depends on type I interferon signaling, as blockade of the IFNAR1 impairs cytotoxic activity despite normal CD8^+^ T cell expansion (Kranz et al. 2016).

However, the strong immune stimulation can sometimes skew responses, leading to autoimmune reactions or an imbalanced immune profile. Consequently, variability in lipid composition and delivery routes can affect the consistency of neutralizing antibody and T-cell responses. While liposome-based mRNA vaccines have demonstrated remarkable efficacy, these challenges highlight the need for continued optimization to minimize adverse effects and enhance therapeutic outcomes (Lee et al. 2023).

### Applications of liposomes as adjuvant for SARS-CoV-2 vaccines

The rapid development of COVID-19 vaccines during 2020–2021 was propelled by advancements in mRNA technology over the past two decades. Unlike traditional vaccines that deliver inactivated or attenuated viruses, mRNA-based vaccines use messenger RNA to instruct human cells to produce the spike protein of SARS-CoV-2, thereby triggering an immune response (Krasnopolsky and Pylypenko 2022). Liposomes played a critical role in this innovation by protecting the fragile mRNA and facilitating its delivery into cells. This approach – exemplified by the Pfizer-BioNTech and Moderna vaccines – set a new standard in biotechnology by achieving high efficacy while minimizing risks such as oncogenesis (Jackson et al. 2020; Krasnopolsky and Pylypenko 2022).

Liposomes composed of ionizable lipids, cholesterol, and other stabilizing molecules are pivotal for ensuring mRNA vaccine stability, transport, and efficacy. Advances in liposome engineering – including novel mixing methods and lipid compositions – enhance vaccine performance by improving antigen presentation and immune activation. For example, the EG-COVID candidate in South Korea has explored lyophilized lipid systems (LSs) to improve storage and distribution conditions (Hong et al. 2021). Another example includes Pfizer-BioNTech and Moderna vaccines, which utilize liposomes as the mRNA vaccine’s adjuvant, licensed and used worldwide, which has been shown in Table 1. In addition to supporting mRNA vaccines, liposomes also enhance the performance of protein subunit vaccines. Numerous liposomal subunit SARS-CoV-2 vaccines are currently under clinical investigation. For example, a ferritin-based vaccine – which self-assembles the SARS-CoV-2 WA-1 spike glycoprotein with a unilamellar liposomal adjuvant containing monophosphoryl lipid A and saponin QS-21 (ALFQ) – elicited robust antibody responses in a Phase 1, first-in-human trial (Ober Shepherd et al. 2024).

Another example is EuCorVac-19 (ECV-19), which uses adjuvanted liposomes combined with the compact receptor binding domain (RBD) of the SARS-CoV-2 spike protein (Mabrouk et al. 2021). Following the recently reported interim Phase 2 trial results, the latest research concluded that the antibody durability of ECV-19 was induced. However, a further Phase 3 trial of ECV-19 is necessary to assess a vast and larger study of the observed antibody responses (Lovell et al. 2024). The development of several SARS-CoV-2 subunit vaccines in a mouse infection model was also being conducted. The first vaccine has a dual TLR ligand liposome adjuvant. It has been proven to protect mice from infection by eliciting local antispike IgA upon challenge (Abhyankar et al. 2021). According to Christensen et al. (2022) that the intranasal boost vaccine (a cationic liposomal adjuvant formulated with Spike Hexa-Pro trimer) was effective against SARS-CoV-2 infection due to the high-magnitude serum-neutralizing antibody responses in the upper respiratory tract of a Syrian hamster model. In addition to this, Ho et al. (2022) formulated a nasal spray vaccine using CpG oligodeoxynucleotides (ODNs) and squalene nanoparticles (PELC) as adjuvants in mice. This approach enhanced APC activation in cervical lymph nodes and significantly improved SARS-CoV-2-specific IgG and IgA antibody production, supporting both systemic and mucosal immunity.

These developments demonstrate the adaptability, versatility, and efficacy of liposome-based and nanoparticle-based delivery systems in modern vaccine design and highlight their critical role in ongoing and future immunization strategies.

The success of liposomal mRNA vaccines during the COVID-19 pandemic has marked a revolutionary advancement in pharmaceutical science. These vaccines achieved efficacy rates of up to 95% and significantly reduced the incidence of severe disease among vaccinated individuals. This innovation has sparked global discussions on the future of biotechnological applications, including cancer immunotherapy and treatments for other infectious diseases. By leveraging the synergistic power of vaccines, nanoparticles, and liposomes, researchers are laying the foundation for next-generation therapeutics. The SARS-CoV-2 pandemic has thus become a defining milestone in the evolution of vaccine technology.

### Opportunities and challenges in liposomal applications

Liposomes are among the most extensively studied nanocarrier systems in drug delivery, owing to their exceptional physicochemical and biological properties (Yan and Huang 2007). One of their most significant advantages is their high biocompatibility, which is attributed to the use of natural or synthetic phospholipids that closely resemble the lipids found in biological membranes (Daraee et al. 2016; Perrie 2008). This structural similarity reduces immunogenicity and cytotoxicity, ensuring that liposomes are biodegradable and safely metabolized within the body. These characteristics make them particularly well-suited for repeated or long-term administration (Haensler 2010; Zamani et al. 2018).

Another crucial advantage is their ability to encapsulate a wide variety of therapeutic agents, including both hydrophilic and hydrophobic molecules (Dimov et al. 2017; Lamichhane et al. 2018; Lombardo et al. 2019; Zununi Vahed et al. 2017). Hydrophilic drugs are retained in the aqueous core of the vesicle, while hydrophobic compounds are incorporated into the lipid bilayer. This dual-loading capacity broadens the range of therapeutic applications, making liposomes especially useful for combination therapy, where multiple drugs with differing solubility profiles must be co-delivered (Riaz et al. 2018; Tyagi et al. 2017; Xing et al. 2016).

In addition, liposomes can be engineered to provide controlled and sustained drug release. By altering the lipid composition, bilayer rigidity, and surface characteristics, researchers can fine-tune the release profile of encapsulated drugs to maintain therapeutic concentrations over extended periods (Gatto et al. 2024; Gregoriadis 2016; Pattni et al. 2015). Such controlled release helps reduce dosing frequency and enhances patient compliance, which is particularly beneficial in chronic disease management (Gonzalez Gomez and Hosseinidoust 2020; Poon and Patel 2020).

Liposomes also significantly improve drug bioavailability, especially for poorly soluble or labile drugs, by shielding them from enzymatic degradation, chemical instability, or rapid clearance from circulation (Alrbyawi et al. 2022; Gonzalez Gomez et al. 2019; Huang and Anderson 2002). Furthermore, their ability to cross biological barriers – such as the BBB – enhances systemic absorption and therapeutic outcomes (dos Santos Rodrigues et al. 2018; Shi et al. 2019).

Another critical advantage of liposomal delivery is the reduction of systemic side effects. Through passive targeting mechanisms – such as the enhanced permeability and retention (EPR) effect in tumors or inflamed tissues – and active targeting via ligand modification, liposomes can preferentially accumulate at the site of disease (Belfiore et al. 2018; Boons 2010; Liu et al. 2020; Noble et al. 2014; Riaz et al. 2018; Wang et al. 2023). This targeted delivery minimizes drug exposure to healthy tissues, thereby reducing off-target toxicity, a common limitation of conventional drug formulations. This property is particularly valuable in oncology and other therapeutic areas requiring high-potency treatments.

Collectively, these advantages highlight the significant potential of liposomes to improve the therapeutic index of drugs, enhance treatment efficacy, and provide safer alternatives in clinical practice (Kim and Jeong 2021; Nsairat et al. 2022).

Despite their promise, liposomes face several challenges that may limit their broader clinical application. One major limitation is stability – liposomes are susceptible to drug leakage, aggregation, and degradation during storage (Gregoriadis 2016; Pasarin et al. 2023). On the manufacturing front, liposome production involves complex, multistep processes that are difficult to scale up while maintaining batch-to-batch consistency in size, encapsulation efficiency, and product quality (Bochicchio et al. 2020; Perrie et al. 2017).

Another concern is immunogenicity, particularly with repeated dosing or cationic liposomal formulations, which can provoke unwanted immune responses or accelerate clearance by the mononuclear phagocyte system (Ochoa-Sánchez et al. 2024; Ponti et al. 2021; Tada et al. 2015). Although PEGylation is widely used to extend circulation time, it can induce the formation of anti-PEG antibodies, contributing to the accelerated blood clearance phenomenon (Mohamed et al. 2019; Nosova et al. 2019; Ren et al. 2019; Shen et al. 2018).

Addressing these issues is essential to fully realizing the clinical utility of liposomal drug delivery systems.

### Future directions and perspectives

The pivotal role of liposomes in the development of SARS-CoV-2 vaccines has underscored their broader potential in medicine (Daraee et al. 2016; Perrie 2008). Future research should focus on optimizing lipid composition, improving liposomal stability, minimizing adverse immune reactions, and advancing large-scale manufacturing techniques. Developing multifunctional liposomes capable of co-delivering both antigens and immunostimulants may further enhance vaccine efficacy and broaden their immunological reach.

In addition, expanding the application of liposomes beyond intramuscular mRNA vaccines to intranasal or oral delivery systems could revolutionize mucosal immunity and improve vaccine accessibility, particularly in low-resource settings. Liposomes also hold immense promise in gene therapy, cancer immunotherapy, and the delivery of CRISPR/Cas9 systems, where precise, targeted delivery is essential to enhance specificity and minimize off-target effects (Zhen et al. 2017; Zhen and Li 2020).

Despite their versatility, key challenges remain, including liposomal stability, immunogenicity, and manufacturing complexity (Gregoriadis 2016; Pasarin et al. 2023). Future efforts should prioritize the design of biodegradable, immune-tolerant liposomal systems that maintain efficacy while reducing toxicity.

Ultimately, sustained interdisciplinary collaboration across immunology, materials science, and nanotechnology will be critical to fully unlocking the potential of liposomes in preventive and therapeutic medicine.

## Conclusions

Liposomes exhibit remarkable versatility and potential in biomedical applications, particularly as adjuvants in vaccine development. Their unique structure and composition make them effective delivery systems that enhance vaccine stability, immunogenicity, and targeted delivery. The success of mRNA vaccines, such as those developed by Pfizer and Moderna for COVID-19, has demonstrated the critical role of liposomal formulations in protecting mRNA, facilitating cellular delivery, and activating robust innate and adaptive immune responses.

Despite these advantages, challenges remain – including the need to optimize lipid compositions, minimize adverse effects, and ensure consistent immune responses across populations. As research advances, liposomes continue to demonstrate strong potential not only as drug delivery systems but also as innovative platforms for enhancing vaccine efficacy and addressing a wide range of infectious diseases and medical conditions.
